# *Pseudomonas aeruginosa* uses multiple receptors for adherence to laminin during infection of the respiratory tract and skin wounds

**DOI:** 10.1038/s41598-019-54622-z

**Published:** 2019-12-03

**Authors:** Magnus Paulsson, Yu-Ching Su, Tamara Ringwood, Fabian Uddén, Kristian Riesbeck

**Affiliations:** 1 Clinical Microbiology, Department of Translational Medicine, Faculty of Medicine, Lund University, Jan Waldenströms gata 59, SE-205 02 Malmö, Sweden; 2 Division for Infectious Diseases, Skåne University Hospital, Lund, Sweden

**Keywords:** Translational research, Infection

## Abstract

*Pseudomonas aeruginosa* efficiently adheres to human tissues, including the lungs and skin, causing infections that are difficult to treat. Laminin is a main component of the extracellular matrix, and in this study we defined bacterial laminin receptors on *P. aeruginosa*. Persistent clinical *P. aeruginosa* isolates from patients with cystic fibrosis, wounds or catheter-related urinary tract infections bound more laminin compared to blood isolates. Laminin receptors in the outer membrane were revealed by 2D-immunblotting, and the specificities of interactions were confirmed with ELISA and biolayer interferometry. Four new high-affinity laminin receptors were identified in the outer membrane; EstA, OprD, OprG and PA3923. Mutated bacteria devoid of these receptors adhered poorly to immobilized laminin. All bacterial receptors bound to the heparin-binding domains on LG4 and LG5 of the laminin alpha chain as assessed with truncated laminin fragments, transmission electron microscopy and inhibition by heparin. In conclusion, *P. aeruginosa* binds laminin via multiple surface receptors, and isolates from lungs of cystic fibrosis patients bound significantly more laminin compared to bacteria isolated from the skin and urine. Since laminin is abundant in both the lungs and skin, we suggest that laminin binding is an important mechanism in *P. aeruginosa* pathogenesis.

## Introduction

*Pseudomonas aeruginosa* is a Gram-negative rod-shaped bacterial species with the ability to cause infections in almost every anatomical compartment of the human body. Opportunistic infections by this species are common in situations with destroyed barriers, such as skin defects in chronic or burn wounds, or epithelial impairment in advanced stages of chronic obstructive pulmonary disease (COPD) or cystic fibrosis (CF)^1,2^.

Most cases of *P. aeruginosa* infections are caused by environmental strains, but strains that cause persistent colonization in patients with CF have adapted significantly and display a changed phenotype. These isolates have characteristically evolved to become more fit for the human host through a series of mutations, exemplified by increased antimicrobial resistance and biofilm mode of growth^3^. We have previously demonstrated that these strains also have gained increased ability to bind vitronectin, a human complement regulator of the terminal pathway of the complement system, and an important component of the extracellular matrix (ECM)^4^.

Several bacterial respiratory pathogens possess strategies to adhere to respiratory epithelial cells or to components of the ECM. This is required in order to prevent trapping and removal by the respiratory mucus, as it is propelled towards the oropharynx by ciliary beating on bronchial epithelial cells^5^. Such bacterial adherence is normally achieved by bacterial surface structures, including pili, flagella or surface-exposed proteins. Persistent *P. aeruginosa* colonization in the lung of patients with CF has been reported to be associated with the ability of bacteria to reside unattached in stagnated mucus^6^. Symptomatic infections by *P. aeruginosa* such as exacerbations in COPD patients, are, however, likely to involve bacterial adhesion to epithelial cells and the ECM.

The ECM is composed of a macromolecule meshwork of glycoproteins making up the adhesion platform for epithelial cells in tissues. It forms a protein scaffold that organizes cells, and it is dynamic and continuously remodeled upon stimuli from surrounding factors^7^. It is mainly composed of proteoglycans such as heparan sulphate, soluble glycoproteins (vitronectin and fibronectin) and fibrous proteins (collagen, elastin and laminin). Intriguingly, *P. aeruginosa* is known to strongly adhere to both desquamated bronchial epithelial cells and to the underlying basal lamina, which is mainly composed of laminin^8^.

The laminin family consists of large ECM proteins (about 800 kDa). These macromolecules are composed of three polypeptide chains (α, β, and γ) that together form an asymmetrical cross-shaped molecule via α-helical coiled coil interactions at their C-terminus. Five laminin globular domains (LG), namely LG1-5, are mapped at the C-terminal end of the alpha-chain (the tips-end of the long arm of laminin molecule). As also seen in other ECM proteins, the laminins have heparin-binding domains (HBDs) that interact with cell-surface bound heparan sulphate^9^.

*Pseudomonas aeruginosa* is associated with ventilator associated pneumonia (VAP) and colonization as well as exacerbations in COPD patients. Smoking, viral infections or mechanical disruption in ventilation may result in inflammatory processes and subsequent damage of the respiratory epithelium, thus exposure of patches of laminin in the basal lamina^10^. Importantly, smokers have thicker laminin layers in their basal lamina, and patients with COPD have increased bronchial deposition of laminin in their respiratory tract. These may offer an ubiquitous availability of ligands for bacterial laminin surface receptors among smokers and COPD patients with inflamed airways^11,12^. The capability of *P. aeruginosa* to bind laminin has been shown, and two unidentified nonpilus outer membrane proteins of 57 and 59 kDa, respectively were suggested as laminin receptors^13^.

We have recently described the *P. aeruginosa* laminin receptor Paf, an orthologue of the laminin-binding *Haemophilus influenzae* Protein F^14^. Paf was discovered through *in silico* analysis based on amino acid sequence similarity with Protein F (PF) of *H. influenzae* and its orthologoues in *Staphylococcus aureus*, *Moraxella catarrhalis*, and group A and B streptococci. We hypothesized that laminin-binding is an important bacterial entity for successful colonization and infection of the human lung, and that the large *P. aeruginosa* genome, in addition to Paf, could potentially contain coding sequences for several laminin receptors. The goal of this study was hence to evaluate the laminin-binding capacity of different clinical *P. aeruginosa* isolates, and to identify laminin receptors at the surface of *P. aeruginosa* strain PAO1 by using a novel and comprehensive proteomic approach.

## Methods

### Bacterial strains and culture conditions

All bacterial strains used in this study are listed in Supplementary Table S1. Twenty-five clinical *P. aeruginosa* isolates were acquired from Clinical Microbiology (Laboratory Medicine, Lund, Sweden) and Clinical Microbiology at Rigshospitalet (Copenhagen, Denmark). All isolates were verified as *P. aeruginosa* by MALDI-TOF. Isolates from patients with CF were selected to represent a persistent strain based on phenotype (mucoidity and antimicrobial susceptibility). Transposon insertion mutants and PAO1 were obtained from the *P. aeruginosa* two-allele library (Washington University, Seattle, WA), and the lack of the expected protein was confirmed with immunoblots using rabbit polyclonal antibodies (pAbs) targeting each protein^15^. Transposon insertion mutants were also verified by DNA sequencing. Bacterial strains were routinely cultured on blood agar plates for 16 hours, or in liquid Lysogeny broth (LB) in 37 °C until OD_600_ = 0.5. Cultures containing *Escherichia coli* with expression vectors were supplemented with appropriate antibiotics.

### Direct binding assay (DBA)

Laminin-111 (L2020; Sigma-Aldrich, St. Louis, MO) was labeled with iodine using the Chloramine-T method^4,14^. Briefly, [^125^I]-laminin was added to an overnight bacterial culture and incubated for 1 h at 37 °C followed by extensive washing steps. Bacterial bound [^125^I]-laminin was measured in a Tri-Carb liquid scintillation counter (Perkin Elmer, Waltham, MA).

### Identification of bacterial laminin receptors by two-dimensional (2D) gel electrophoresis

Bacterial OMP fractions were prepared from overnight cultures as described in detail^16^. Briefly, bacterial membranes were pelleted by ultracentrifugation at 150,000 × g at 4 °C for 1 h followed by 2D-sodium dodecyl sulfate–polyacrylamide gel electrophoresis (SDS-PAGE) and blotting. For detection of laminin-binding proteins, membranes were incubated with Engelbreth-Holm-Swarm murine sarcoma laminin-111 (Sigma-Aldrich) and detected with rabbit anti-mouse laminin-111 polyclonal antibodies (pAb) (Sigma-Aldrich). Horseradish peroxidase (HRP)-conjugated swine anti-rabbit pAb (Dako, Glostrup, Denmark) were used as a secondary antibody. Images of immunoblots were aquired in parallel with the appropriate control at the same exposure time. Contrast and brightness were adjusted in Photoshop (Adobe Systems, San Jose, CA) and applied equally over the entire image to harmonize grey backgrounds between experiments and controls.

### Expression and purification of recombinant proteins

The open reading frames (ORF) of the potential bacterial laminin receptors were amplified from PAO1 genomic DNA with restriction site specific primers (Supplementary Table S2), followed by cloning into pET26(b) + (Novagen, Darmstadt, Germany). The DNA sequences of all plasmids were verified with sequencing (Eurofins Genomics, Ebersberg, Germany). *Escherichia coli* BL21(DE3) transformed with the appropriate recombinant DNA plasmid constructs was used for protein expression of 6 × His-tagged bacterial recombinant proteins. The recombinant proteins were purified by affinity chromatography using Ni-NTA resin (GE Healthcare, Chicago, IL), as previously described^17^. His-tagged laminin LG domains were expressed and purified as described^16^.

### Production of rabbit polyclonal antibodies (pAb)

Rabbits were immunized intramuscularly with 200 μg of each of the recombinantly expressed potential bacterial laminin receptors, emulsified in complete Freund’s adjuvant (Difco, Becton Dickinson, Heidelberg, Germany), and were boosted on days 18 and 36 with the same dose of protein in incomplete Freund’s adjuvant. Blood was drawn 2 to 3 weeks later. Immunoblots were performed to ensure that each antiserum reacted with the expected recombinantly expressed protein. Resulting pAbs were purified using affinity chromatography.

### Enzyme-linked immunosorbent assay (ELISA)

To analyze interactions between *P. aeruginosa* OMPs and laminin-111, laminin-111 at concentrations of 0.078 nM–5 nM were coated in 96-well PolySorp plates (Thermo Fisher Scientific, Waltham, MA) in coating buffer (100 mM Tris-HCl, pH 9.0) for overnight at 4 °C. Following a standard ELISA protocol, ligands (recombinant bacterial proteins; 50 nM-100 nM) were added in PBS-0.05% Tween-20 with 1% fish gelatin and bound bacterial proteins were detected using HRP-conjugated anti-His pAbs at dilution 1:5,000. Absorbance was read at 450 nm. The IgD-binding region of *Moraxella catarrhalis* IgD binding protein (MID 962–1200), which does not bind laminin, was included as a negative control^18^. For heparin inhibition, 2.5 nM laminin-111 was coated in microtiter plates and ligands were added together with heparin at 0.1–1000 μg/ml. For analysis of LG domains (1–3, 4 or 5), 0.125–8 μg/ml of the recombinant LG were coated as above. Bound ligands were detected using rabbit pAbs directed to each bacterial protein analysed, and HRP-conjugated swine anti-rabbit pAbs.

### Biolayer interferometry and calculation of affinity constants

Analysis of the kinetics of bacterial-laminin binding was performed by biolayer interferometry (BLI) using a forteBio OctetRed96 (Pall, Menlo Park, CA). The potential *P. aeruginosa* laminin receptors were immobilized on AR2G sensors (Pall) by amino coupling. The analyte (laminin-111) was serially diluted in running buffer (PBS) ranging from 1.63 to 100 nM after an initial pH loading screen. The experiments were conducted at 30 °C. Data analysis was performed using the forteBio Data Analysis software 8.1 (Pall) and the K_ass_, K_diss_, and affinity (K_D_) were calculated.

### Binding of *P. aeruginosa* to immobilized laminin-111

Eight-well, 6 mm diagnostic microscope slides (Thermo Fisher scientific) were coated with 0.5 μg laminin-111 or 5 μg BSA in 25 μl PBS followed by airdrying and washing in PBS. Twenty-five μl of a bacterial culture (OD_600_ = 0.5) was added to slides and incubated at 37 °C for 1 h. After washing steps and drying, the samples were Gram-stained and examined at 40 × in a light microscope. Adhered bacterial cells were counted in 3 biological replicates by an automated image analysis (counting tool) in cellSens Dimensions v 1.9 (Olympus Life Science, Tokyo, Japan) in 6 randomly chosen regions of interests (ROI) on the slide, each with an area of 10^5^ μm^2^, to minimize observer bias.

### Transmission electron microscopy (TEM)

The interaction of bacterial surface proteins with laminin was visualized by negative staining and TEM. Laminin and putative laminin-binding receptors were labeled with colloidal gold for visualization in TEM. Equal volumes of labeled laminin (10 μg/ml) were incubated with gold-labeled purified protein (5 μg/ml) for 1 h at 37 °C according to standard protocols^19^. Specimens were examined in a Philips/FEICM 100 TWIN transmission electron microscope operated at 60 kV accelerating voltage by Colzyx (Lund, Sweden). Images were recorded with a side-mounted Olympus Veleta camera with a resolution of 2048 × 2048 pixels and the iTEM acquisitions software (Olympus Life Science).

### Statistical analyses

Statistical differences were analyzed using one-way ANOVA, with Tukey’s or Dunnett’s multiple comparison test as recommended for the dataset by the software, or multiple *t*-test with Holm-Sidak correction for multiple comparison (Prism, GraphPad Software, La Jolla, CA). For all experiments, *p*-values ≤ 0.05 were considered statistically significant.

### Ethics

Experiments utilizing animals were approved by the Swedish Board of Agriculture (Lund and Malmö Tingsrätt dnr. 4438/2017) and all experiments were performed in accordance with relevant guidelines and regulations set up by this institution.

## Results

### *Pseudomonas aeruginosa* isolated from CF patients have adapted to bind laminin

The laminin-binding capabilities of clinical *P. aeruginosa* isolates were interrogated using [^125^I]-labelled laminin-111 in a DBA. Clinical isolates were collected from sputum from patients with CF (*n* = 6), catheter-related urinary tract infection (*n* = 6), and wound infection (*n* = 6). In addition, we analysed *P. aeruginosa* blood isolates from patients with acute bacteremia (*n* = 7). Some of the isolates collected from the urinary tract or wounds where from chronically infected patients. Isolates from sputum of CF patients were considered as persistent, whereas the bacteremia isolates normally represent nonclonal, unadapted environmental strains^20^.

**Figure 1:**
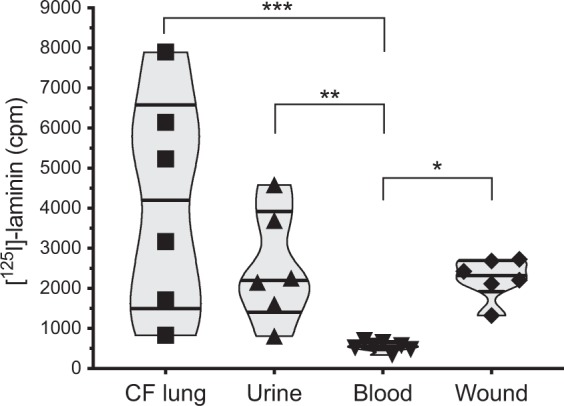
Clinical strains from persistent infections bind laminin. Twenty-five clinical *Pseudomonas aeruginosa* strains were examined in a direct binding assay (DBA) comprising [^125^I]-laminin 111. Radioactivity was recorded in a liquid scintillation counter after unbound laminin was removed by thorough washing. The violin graphs show median and quartiles, and each isolate from the lung of patients with cystic fibrosis (CF, squares), patients with urinary tract infections (triangles), bacteremia (down-facing triangle) and persistent wound infections (diamonds) as indicated. cpm; counts per minute. *p ≤ 0.05; **p ≤ 0.01; ***p ≤ 0.001. Statistical significances were calculated using one-way ANOVA with Tukey’s multiple comparison test.

Interestingly, the highest laminin-binding capacity was found among the *P. aeruginosa* sputum isolates from patients suffering from CF (Fig. 1). The mean [^125^I]-laminin-binding by these bacterial strains was 7.5-fold higher than isolates from blood samples (*p* < 0.001), and also somewhat higher than isolates from urine samples or wounds, albeit not statistically significant. In general blood isolates did not exhibit a strong binding to laminin, and had a 3.9-fold lower laminin-binding capacity compared to urine (*p* = 0.003) and a 4.1-fold lower binding capacity than wound isolates (*p* = 0.014).

### The *P. aeruginosa* laminin receptors are defined by 2D-SDS-PAGE and immunoassay

**Figure 2:**
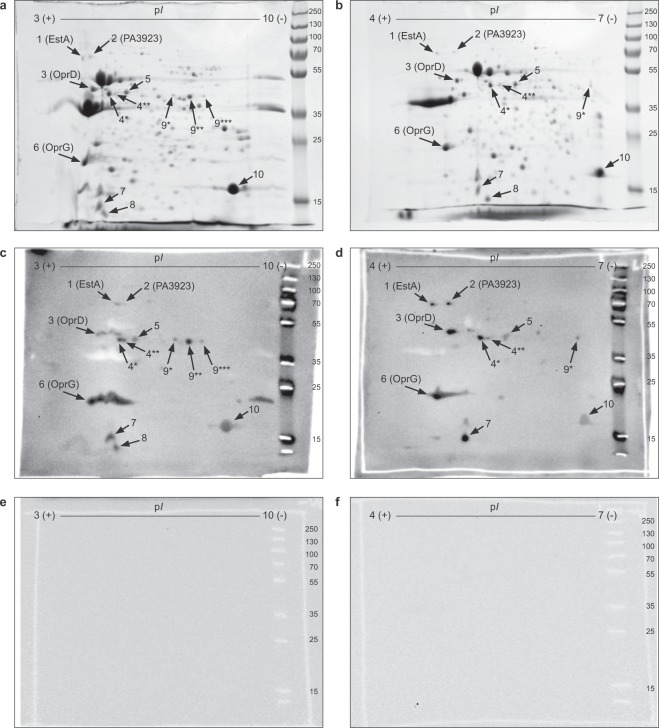
Proteomic identification of *P. aeruginosa* proteins that interact with laminin. Two-dimensional (2D) separation of outer membrane proteins from *P. aeruginosa* PAO1 by 2D-SDS-PAGE (**a**,**b**), and electrotransferred onto membrane blots that were subsequently incubated with laminin-111, rabbit anti-laminin pAbs and HRP conjugated swine anti-rabbit pAbs (**c**,**d**). Panels e and f comprise the background control of primary and secondary pAbs. Laminin-binding protein spots were excised from gels and identified by sequencing using MALDI-TOF, of which four were confirmed laminin-binding outer membrane proteins, *i.e*. EstA, OprD, OprG and PA3923. Numbers and arrows point toward each identified protein. The numbering and asterixis correspond to Table S3 in supplementary data. Denoted numbers to the right of the protein ladder in each picture indicate size in kilodalton. pI and numbers denoted at the top of each picture indicate the isoelectric point. All images are of full-length gels or immunoblots. Brightness and contrast have been adjusted over entire images to harmonize background levels.

A proteomic approach was used to identify laminin-binding proteins in the outer membrane of the reference strain *P. aeruginosa* PAO1. The OMPs of PAO1 were purified and separated by 2D-SDS-PAGE (Fig. 2a,b). A large number of spots (*n* = 194) were visible in the coomassie stained gel. By using laminin-111 as a bait, we identified 13 laminin-binding signal spots (including multiple isoforms) which represented 10 discrete putative laminin-binding proteins (Fig. 2c,d). These were excised from gels and subjected to MALDI-TOF analysis. Smearing artifacts or horizontal streakings observed on some protein spots could be attributed to, (i) post-translational modifications, or/and (ii) incomplete focus or overfocusing of protein species that were highly abundant in the sample^21^. The full list of identified proteins is available in Supplementary Table S3. Eight proteins had an amino acid sequence indicating localization in the outer membrane^22^. In addition to Paf that has previously been identified as a laminin-receptor on *P. aeruginosa*^14^, four of those, *i.e*., EstA, OprD, OprG and PA3923, were further characterized as laminin-receptors in downstream analysis. The remaining six putative laminin-binding proteins were discarded either since the amino acid sequence indicated other subcellular localization than the outer membrane, or since laminin-binding could not be confirmed in downstream experiments.

### Transposon mutants lacking functional EstA, OprD, OprG, PA3923 or Paf have a reduced laminin-binding capacity

**Figure 3:**
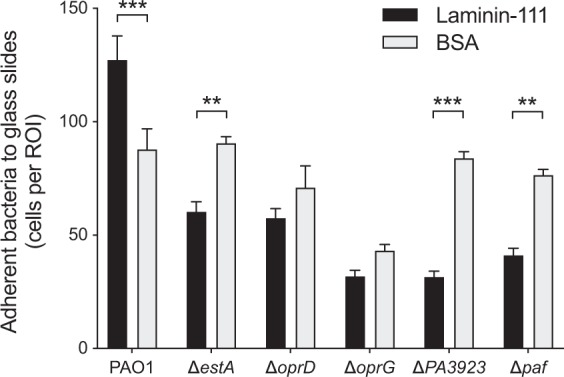
Each laminin-receptor contribute to the laminin-adhering phenotype. *Pseudomonas aeruginosa* mutants from a transposon library were examined for adherence to laminin-111 coated on glass slides (mean and standard error mean (SEM)). Bovine serum albumin (BSA) was used for negative control. *P*-values shown indicate the statistical difference of the means of adherent bacteria to laminin- versus to BSA-coated slides, as evaluated with repeated *t-*test and correction for multiple comparisons using the Holm-Sidak method. Adherent bacteria were automatically counted by the image analysis software cellSens in 6 randomly selected regions of interest, in biological triplicates. *p ≤ 0.05; **p ≤ 0.01; ***p ≤ 0.001. Representative images for wild type PAO1 and each mutated strain as denoted are shown in the supplemental data, Fig. S1.

To confirm that the laminin-binding proteins identified in the 2D-SDS-PAGE were not due to an experimental artefact, transposon insertion mutated *P. aeruginosa* were examined for their laminin-binding phenotypes. Since laminin is mainly located in the basal lamina, we immobilized laminin on glass slides and added the wild type PAO1 strain or corresponding transposon mutants lacking one of the laminin receptors. Adherent bacteria were counted after extensive washing steps. Even though only one protein was devoid in each mutated strain, the functional laminin-binding was reduced in all mutated strains compared to the wild type (all *p* < 0.001, one-way ANOVA with Dunnet’s multiple comparisons test). However, to ensure that the general adhesive phenotypes were unchanged in the transposon mutants, we subjected bacteria to albumin-coated glass-slides in parallel to laminin. Only the wild type strain (PA01) adhered better to laminin than to albumin, while all tested *P. aeruginosa* mutants adhered better to albumin-coated than to laminin-coated glass-slides (Figs. 3 and S1).

### Recombinant EstA, OprD, OprG, PA3923 and Paf interact with laminin with high affinity

**Figure 4:**
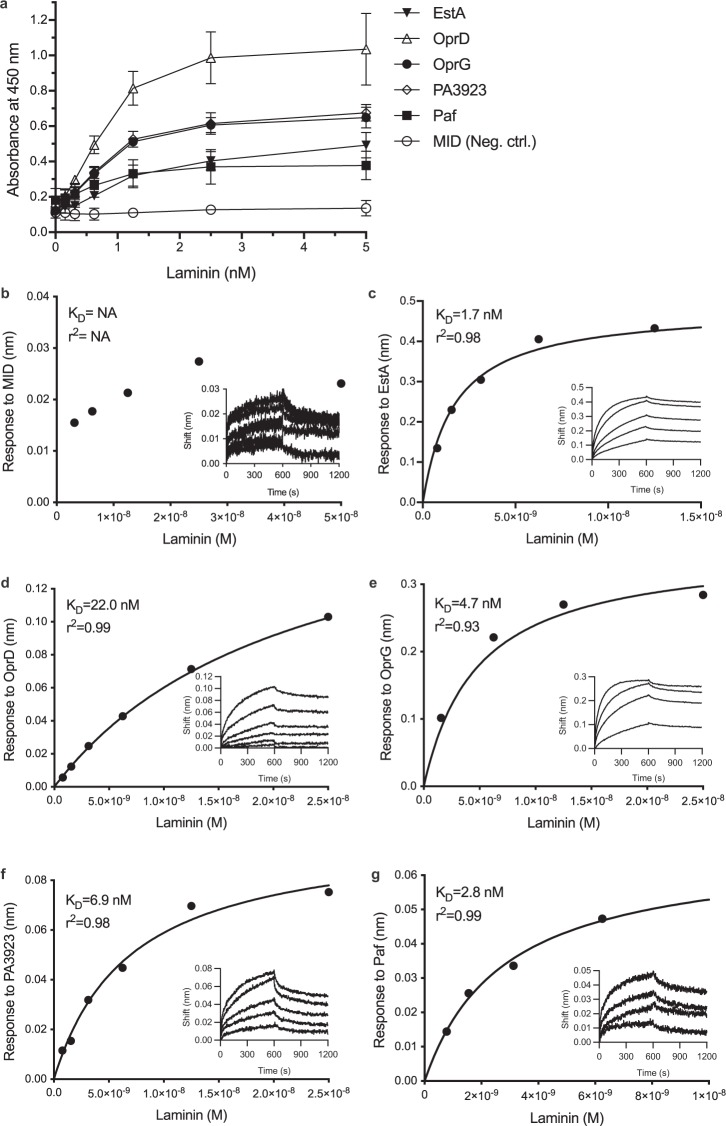
*Pseudomonas aeruginosa* receptors bind laminin with wide range of affinities. Recombinantly expressed proteins EstA, OprD, OprG, PA3923 and Paf, purified with affinity chromatography, were subjected to laminin-coated plates and binding was detected by ELISA using anti-His pAbs. In addition, Moraxella IgD-binding protein (MID), was added to the experiments as negative control. Three biological repetitions were done. The binding to laminin of each of the four outer membrane proteins and Paf were dose-dependent up to saturation **(a)**. The interactions were further evaluated with biolayer interferometry (**b–f**) and dissociation constants (K_D_) calculated from the fitting model.

To gather additional details on the interaction between laminin and the putative bacterial receptors, selected *P. aeruginosa* OMPs were recombinantly expressed and purified using affinity chromatography. When analysed by ELISA, we found that EstA, OprD, OprG, PA3923 and Paf bound to laminin in a dose-dependent manner, and saturation was achieved indicating specific binding (Fig. 4a). To evaluate the kinetics of interactions, we performed a biolayer interferometry assay with the putative laminin receptor immobilized to the sensors (Fig. 4b–g). This experiment revealed that all five *Pseudomonas* laminin-receptors bound to laminin albeit with a wide range of affinities (EstA K_D_ = 1.7 nM; OprD K_D_ = 22.0 nM; OprG K_D_ = 4.7 nM; PA3923 K_D_ = 6.9 nM; Paf K_D_ = 2.8 nM). MID that was used as negative control, did not bind to laminin in ELISA, nor in the interferometry assay.

### EstA, OprD, OprG, PA3923 and Paf bind to the laminin globular domains LG4 and LG5, and the interactions are inhibited by heparin

It has previously been shown that bacterial receptors bind to HBDs on ECM proteins^5^. The LG domains (LG4 and LG5) at the C-terminal of the α-subunit contain HBDs, and are hence predicted as the binding site for these bacterial receptors^23^.

**Figure 5:**
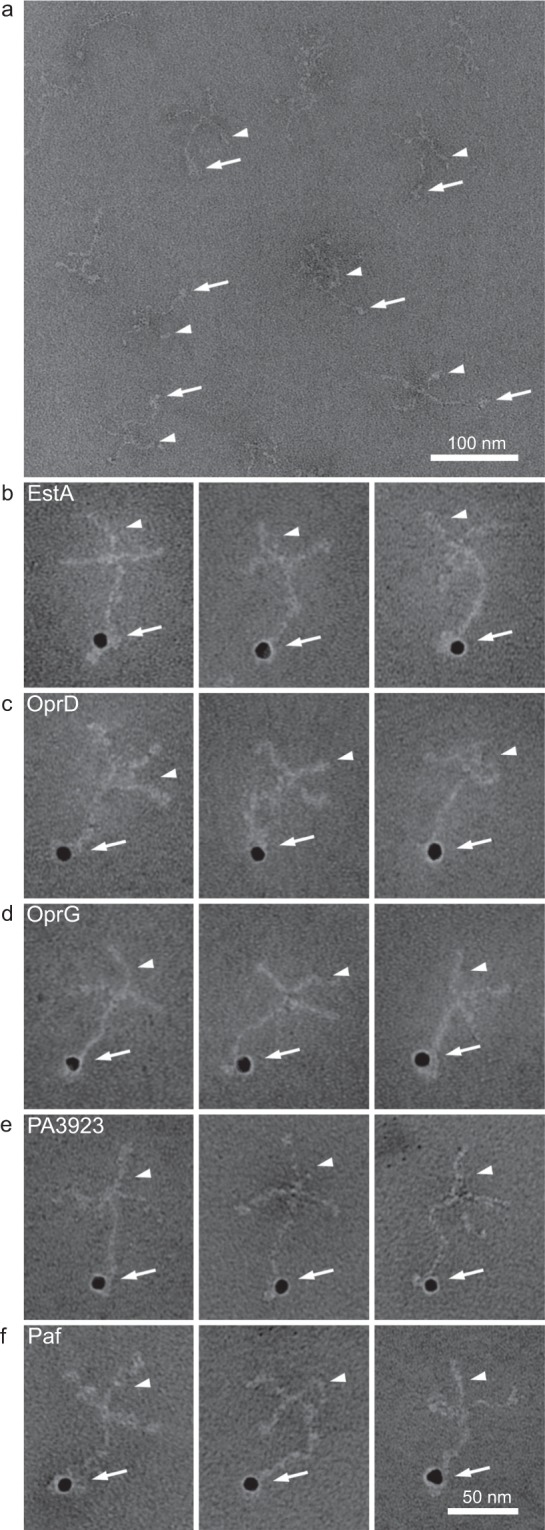
The *Pseudomonas aeruginosa* laminin-receptors bind to the globular domains. Laminin-111 was visualized by TEM (**a**) The scalebar indicates 100 nm, and arrows point at the LG domain at the C-terminal of the alpha-subunit, whereas arrowheads mark the short arms of the alpha, beta and gamma subunits. Laminin (arrow heads) was incubated with EstA (**b**), OprD (**c**), OprG (**d**), PA3923 (**e**) and Paf (**f**), which were labelled with 10 nm colloidal gold (arrows). All bacterial receptors adhered to the LG domains at the base of the cross-shaped laminin molecule. Three representative pictures of each protein are shown, and the scale bar indicates 50 nm.

**Figure 6:**
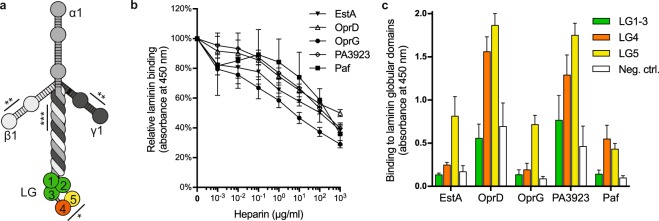
The heparin-binding domains at LG4 and LG5 are targeted by *P. aeruginosa* laminin-binding proteins. Schematic cartoon of laminin-111 with the alpha (α, medium grey), beta (β, light grey) and gamma (γ, dark grey) subunits forming a cross-shaped molecule (**a**). The five laminin globular (LG) domains at the base of the molecule are coloured (green, LG1-3; orange, LG4; and yellow, LG5). Heparin-binding sites are denoted with line and asterisks (* = hep-1, ** = hep-2 and *** = hep-3)^16^. Interactions between the bacterial receptors and laminin were found to be inhibited by preincubation with increasing concentrations of heparin. This indicated the involvement of HBDs (**b**). Truncated fragments of LG (LG1-3, LG4 and LG5) were recombinantly expressed and the interactions with the *P. aeruginosa* receptors were analyzed by ELISA using rabbit pAbs for each bacterial receptor (**c**). In negative controls (Neg. ctrl.), the ligands were omitted. All results are presented as the mean values from three independent repetitions. Error bars represent standard deviation of the data.

The typical cross-shaped laminin molecule is easily recognized in TEM. This was exploited to determine the binding site for each bacterial recombinant protein (Fig. 5a). All tested OMPs adhered to the tips of the long arm of the laminin molecules, which is where LG4 and LG5 are located (Figs. 5b–f and 6a). Involvement of HBDs was further indirectly supported by a heparin inhibition assay. Preincubation with increasing concentrations of heparan sulphate prior to ELISA gradually repressed the interaction between each of the five *P. aeruginosa* OMPs and laminin (Fig. 6b).

There are, however, additional HBDs on the laminin molecule, *i.e*. “hep-2” and “hep-3”, on the short arms and the centre of the cross, respectively^24^. To gain evidence of where on laminin these bacterial OMPs adhered, LG domains (LG1-3, LG4 and LG5) were recombinantly expressed followed by purification by affinity chromatography. Polyclonal rabbit antibodies were produced for each *P. aeruginosa* OMP, and used to determine binding in ELISA. As seen in Fig. 6c, OMP-dependent binding was generally strongest to LG5 and, in the case of OprD, PA3923 and Paf, binding could also be observed to LG4, whereas the binding to LG1-3 did not differ significantly from the negative control. Hence, all five *Pseudomonas* laminin-receptors bound to LG5 and three of five bound also to LG4.

## Discussion

In this study, we report that clinical strains of *P. aeruginosa* bind laminin to the bacterial surface. In addition, we have identified four OMP as new laminin-receptors on *P. aeruginosa*, that is, EstA, OprD, OprG and PA3923. The abundance of receptors indicates that laminin-binding is of high clinical importance, but complicates the interpretation of the biological importance of individual receptors. From previous experiments it is a well known fact that mutated bacteria lacking one major membrane protein upregulate other membrane proteins. This can be exemplified by a study by Skurnik *et al*. in which mutated *P. aeruginosa* PA14 lacking *oprD* were found to have a more than 10-fold increased expression of *oprG* and *oprQ*^25^. In addition, due to the expected high biological importance of laminin binding for bacterial infection, it cannot be excluded that *P. aeruginosa* in its large genome possesses additional sequences encoding laminin receptors. Despite this, in the present study several of the mutated strains lacking only one receptor had a poor laminin-binding phenotype.

Direct and indirect evidence suggests involvement of laminin binding to the heparin-binding domains on LG4 and LG5 on the tips of the long arm of the laminin alpha chain molecule, but it cannot be excluded that other heparin-binding sites are involved on the laminin molecule. The involvement of HBDs is in accordance with most previous observations of bacterial receptor – human ECM protein interactions, and forms a potential therapeutic target for heparin or heparin-derived compounds during infections^5^.

Bacterial isolates that have adapted to the human host during long term colonization in the lungs bound significantly more laminin than isolates from patients with bacteremia. We have previously reported a corresponding finding on vitronectin-binding by *P. aeruginosa*^4^. Vitronectin was more efficiently attracted to the surface of *P. aeruginosa* CF isolates than to bacterial isolates obtained from blood samples. Typically, adapted *P. aeruginosa* CF isolates have evolved phenotypic traits that are favourable for colonization of the respiratory tract^3^. Since improved ECM binding is repeatedly observed in adapted strains, we speculate that adhesion to epithelial cells or to exposed basal lamina is beneficial to avoid expulsion by the mucociliary escalator at several stages of the infection.

All identified laminin-receptors bound laminin with high affinity (K_D_ 1.7–22.0 nM) in biolayer interferometry. Each identified receptor is discussed in the following sections ordered after affinity for laminin. The biological importance of each receptor is, however, also dependent on other factors, such as density of a specific receptor at the bacterial surface.

EstA is an autotransporter, and has been described as an excreted catalytic enzyme that is important for *P. aeruginosa* virulence in a rat model of chronic respiratory infection^26^. Interestingly, EstA bound to laminin with the highest affinity (Kd = 1.7 nM). Based on its signal peptide, it is predicted to be located in the outer membrane with the N-terminal exposed, though it has been observed in both the periplasm and outer membrane vesicles (OMVs) of *P. aeruginosa*^27–29^. Our observations support localization of EstA in the outer membrane, and add on the laminin interaction to the previously suggested functions.

In our experiments, Paf was the *P. aeruginosa* receptor with the second highest affinity for laminin (K_D_ = 2.8 nM). We have previously identified laminin-binding properties of Paf, as well as of the orthologues *Moraxella catarrhalis* AfeA, *Staphylococcus aureus* MntC and *Haemophilus influenzae* PF^14^. Paf is an ABC-transporter, and as such Paf was not encountered in the 2D immunoblotting of the OMP preparation.

OprG is one of the main porins in *P. aeruginosa*, and the laminin receptor with the third highest affinity in the present study (K_D_ = 4.7 nM). This small (25.2 kDa) beta-barrel shaped protein has loops that extend outwards from the outer membrane and functions by Fe^2+^ acquisition^30^. OprG belongs to the OmpW-family of membrane proteins that is shared among many Gram-negative bacterial species. OmpW in *Burkholderia cepacia* was recently shown to be involved in attachment to respiratory epithelial cells, but the present study is the first report of an ECM-binding OmpW-family member^31^.

The *P. aeruginosa* protein derived from gene PA3923 is a previously hypothetical protein with unknown function in the bacterial cell. In the present study, it was identified and characterized as a laminin-receptor with high affinity for laminin (K_D_ = 6.9 nM). Based on the amino acid sequence, PA3923 has a molecular weight of 69.6 kDa and it contains a signal peptide for localization in the outer membrane. According to the UniProt database, it belongs to a group of DUF1302 domain-containing proteins. Most other proteins in this group belong to the *Pseudomonas* family, but *Acinetobacter baumannii* and *Enterobacter cloacae* are also represented.

Finally, in this study, OprD (K_D_ = 22.0 nM) was the laminin-receptor with the lowest affinity. OprD (porin D, OccD1) is best known as a mutated imipenem channel causing carbapenem resistance in clinical isolates. This beta-barrel shaped protein forms a narrow basic amino acid channel, with external loops^30^. We have previously described OprD as a vitronectin receptor, which affects the bacterial ability to resist complement proteins of the innate immunity^4^. The OprD superfamily also includes OprQ, which has previously been identified as a fibronectin receptor^32^. Since OprD interacts with the HBDs both on laminin and on vitronectin, a common binding site on OprD is suggested, which needs to be further explored.

In conclusion, in the present study we used a global proteomic approach to identify four new laminin receptors in *P. aeruginosa*, in addition to confirm and further characterize Paf as a laminin receptor. We found that lung isolates derived from CF patients were capable laminin-binders, and since laminin is a main component of the pulmonary ECM, our study suggests mechanistic insights in the pathogenesis of *P. aeruginosa* lung infection. In addition, our results support the idea of a therapeutic antibacterial strategy based on the inhibitory effect of heparin on the interaction between bacteria and human ECM proteins, an approach that warrants further exploration in a clinical setting.

## Supplementary information

Supplementary data

## Data Availability

The datasets generated during and/or analysed during the current study are available from the corresponding author on reasonable request.
