# Supplementary material for: Pseudomonas aeruginosa uses multiple receptors for adherence to laminin during infection of the respiratory tract and skin wounds
Source: Sci Rep. 2019 Dec 3;9:18168. doi: 10.1038/s41598-019-54622-z (PMC6890786; doi:10.1038/s41598-019-54622-z)
Supplement: Supplementary file 1 — Supplementary data [file 41598_2019_54622_MOESM1_ESM.pdf]

Supplementary data

Pseudomonas aeruginosa uses multiple receptors for adherence to laminin during infection of the respiratory tract and skin wounds

Magnus Paulsson[a, b], Yu-Ching Su[a], Tamara Ringwood[a], Fabian Uddén[a] and Kristian Riesbeck[a]

[a] Clinical Microbiology, Department of Translational Medicine, Faculty of Medicine, Lund University, Jan Waldenströms gata 59, SE-205 02 Malmö, Sweden

[b] Division for Infectious Diseases, Skåne University Hospital, Lund, Sweden

Supplementary data, Table S1: Clinical and laboratory strains used in this study

Clinical strains

Name	*Description*	*Reference*
KR796, KR797, KR801, KR802, KR809, KR811	Clinical *P. aeruginosa* isolates from airway cultures of patients with CF	[1]
KR794, KR798, KR825, KR826, KR827, KR828	Clinical *P. aeruginosa* isolate from urine culture	[1] and this study
KR799, KR800, KR812, KR813, KR814, KR829, KR830	Clinical *P. aeruginosa* isolates from blood cultures	[1] and this study
KR1158, KR1159, KR1160, KR1161, KR1162, KR1163	Clinical *P. aeruginosa* isolates from chronic wound cultures	This study

Laboratory strains

Name	*Description*	*Reference*
E. coli BL21(DE3)	*E.coli* laboratory strain	
E. coli-EstA	*E.coli* BL21(DE3) with vector pet-26(b)+ and insert *estA*	This study
E. coli-OprD	*E.coli* BL21(DE3) with vector pet-26(b)+ and insert *oprD*	[1]
E. coli-OprG	*E.coli* BL21(DE3) with vector pet-26(b)+ and insert *oprG*	This study
E. coli-PA3923	*E.coli* BL21(DE3) with vector pet-26(b)+ and insert *PA3923*	This study
E. coli-Paf	*E.coli* BL21(DE3) with vector pet-26(b)+ and insert *paf*	[2]
MPAO1	*P. aeruginosa* reference strain. Clone of PAO1.	[3]
PW9583	MPAO1 mutant with transposon insert in *estA* (PA5112)	[3]
PW2742	MPAO1 mutant with transposon insert in *oprD* (PA0958).	[3]
PW7877	MPAO1 mutant with transposon insert in *oprG* (PA4067)	[3]
PW7631	MPAO1 mutant with transposon insert in loci PA3923	[3]
PW5054	MPAO1 mutant with transposon insert in *paf* (PA2407)	[3]

Supplementary data, Table S2: List of primers used for cloning.

Primer	Sequence 5'-3'	GenBank Accession number	Locus tag
*estA*_fwd[a]	GAAC*GGATCC*GGCTCCTTCGCCCTATTCGACG	AAG08497.1	PA5112
*estA*_rev[b]	TCAG*AAGCTT*GAAGTCCAGGCTCAGCGCCAG		
*oprD*_fwd[a]	CTGA*GGATCC*GGACGCATTCGTCAGCGATCAG GCC	AAG04347.1	PA0958
*oprD*_rev[b]	CTGAC*AAGCTT*CAGGATCGACAGCGGATAGTC GACGATCAG		
*oprG*_fwd[a]	GAAC*GGATCC*GGCGGATATTCAAGGACACAAG G	AAG07454.1	PA4067
*oprG*_rev[b]	TCAG*AAGCTT*GAACTTGTAGCCGAAACCGATCA		
*PA3923*_fwd[a]	GAAC*GGATCC*GGTGACCTTCAACATCGGGGAA AT	AAG07310.1	PA3923
*PA3923*_rev[b]	TCAG*AAGCTT*GAAGTTCATGCCGAAGCTGAG		
*Paf*_fwd[a]	AA*GGATCC*GGAAGACGGCAAACGCCTG	AAG05795.1	PA2407
*Paf*_rev[b]	TT*AAGCTT*TGCGCCGGACTCCTGGATG		

[a-b] Restriction enzyme cutting sites are in italic type: [a]*Bam*HI, [b]*Hin*dIII

Supplementary data, Table S3: List of identified proteins by MALDI-TOF in 2D-SDS PAGE.

ID[a]	Protein description and function	Gene[b]	Locus tag[b]	GenBank accession number[c]	Molecular weight on the gel (kDa)	Predicted molecular weight (kDa)[d]	pI[d]	Subcellular localization[e]	Score	Sequence coverage (%)
1	Autotransporter esterase	*estA*	PA5112	AAG08497	70	69.6	4.68	Outer membrane	394	37
2	Hypothetical protein	-	PA3923	AAG07310	72	69.5	4.94	Outer membrane	270	28
3	Porin D	*oprD*	PA0958	NP_249649	48	48.4	4.96	Outer membrane	665	73
4*	outer membrane porin OprQ	*oprQ*	PA2760	AAG06148	45	46.9	5.54	Outer membrane	736	60
4**	outer membrane porin OprQ	*oprQ*	PA2760	AAG06148	45	46.9	5.80	Outer membrane	474	55
5	FadL long chain fatty acid transport protein	*fadL*	PA1288	AAG04677	45	45.6	5.73	Outer membrane	557	53
6	Outer membrane protein OprG	*oprG*	PA4067	AAG07454	25	25.2	4.85	Outer membrane	375	50
7	Lipid A 3-O-deacyllase, PagL	*pagL*	PA4661	AAG08048	20	18.4	5.86	Unknown	449	100
8	DNA-binding stress protein	*dps*	PA0962	AAG04351	15	17.5	4.96	Cytoplasmic	410	69
9*	anaerobically-induced outer membrane porin OprE	*oprE*	PA0291	AAG03680	40	49.7	7.00	Outer membrane	502	59
9**	anaerobically-induced outer membrane porin OprE	*oprE*	PA0291	AAG03680	40	49.7	8.67	Outer membrane	722	71
9***	anaerobically-induced outer	*oprE*	PA0291	AAG03680	40	49.7	8.80	Outer membrane	372	49

	membrane porin OprE								
10	PhoP/Q and low Mg2+ inducible outer membrane protein H1	*oprH*	PA1178	AAG04567	16	21.6	9.00	Outer membrane	614	74

[a] Spot 4* and 4** are the isoforms (different pI but same molecular weight) of OprQ; spot 9*, 9** and 9*** are the isoforms of OprE.
[b] Gene name and locus tag are based on Pseudomonas Genome DB http://pseudomonas.com/
[c] Accession number indicated is based upon the PAO1 annotated genome database at GenBank.
[d] Molecular weight and isoelectric focusing point (pI) of each protein is predicted using Protparam tool (https://web.expasy.org/protparam/) except for isoform spots 4**, 9* and 9*** that were estimated from the gels.
[e] Predicted subcellular localization based on PSORTb v3.0.

Supplementary data, Figure S1

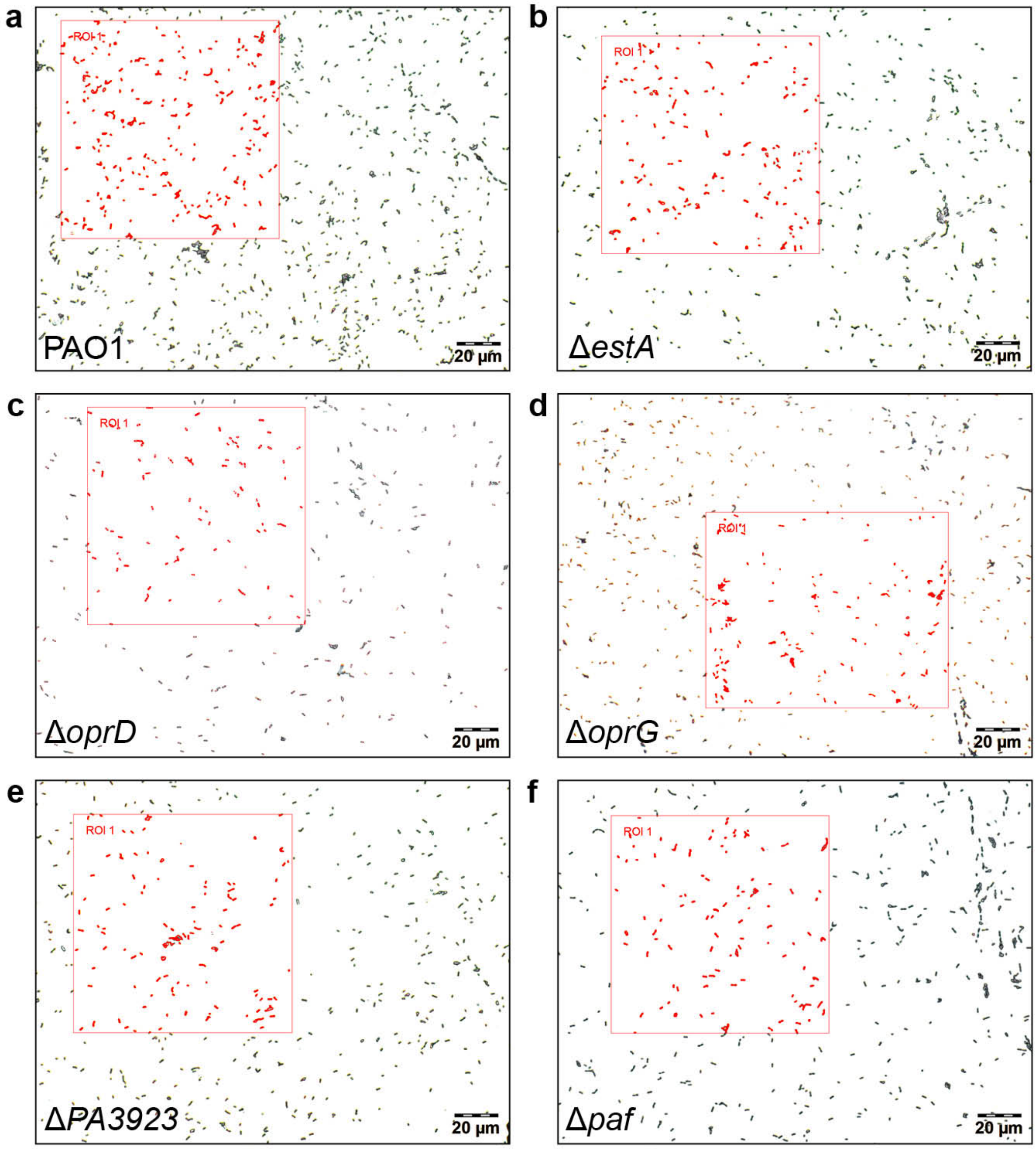

Figure S1. ***Pseudomonas aeruginosa*** **adherence to immobilized laminin**. *Pseudomonas aeruginosa* wild type PAO1(**a**) and mutated strains devoid of laminin receptors (**b**-**f**, as denoted) were subjected to laminin-111 coated glass slides. Adherent bacteria were automatically counted by the light microscopy image analysis software cellSens (Olympus) in 6 randomly selected regions of interest (ROI, red), in biological triplicates. One representative picture of each strain is shown.

References

1. Paulsson, M. *et al.* Identification of outer membrane Porin D as a vitronectin-binding factor in cystic fibrosis clinical isolates of *Pseudomonas aeruginosa*. *J. Cyst. Fibros.* **14,** 600–607 (2015).
2. Su, Y.-C. *et al. Haemophilus* Protein F Orthologs of Pathogens Infecting the Airways: Exploiting Host Laminin at Heparin-Binding Sites for Maximal Adherence to Epithelial Cells. *J. Infect. Dis.* **216,** 1303–1307 (2017).
3. Held, K., Ramage, E., Jacobs, M., Gallagher, L. & Manoil, C. Sequence-verified two-allele transposon mutant library for *Pseudomonas aeruginosa* PAO1. *J. Bacteriol.* **194,** 6387–6389 (2012).
